# The impact of interleukin-10 (IL-10) gene 4 polymorphisms on peripheral blood IL-10 variation and prostate cancer risk based on published studies

**DOI:** 10.18632/oncotarget.17522

**Published:** 2017-04-29

**Authors:** Tingting Men, Cuicui Yu, Dan Wang, Fang Liu, Jingjing Li, Xiaoying Qi, Chunhua Yang, Wenguo Jiang, Xiaodan Wei, Xuri Li, Bin Wang, Jia Mi, Geng Tian

**Affiliations:** ^1^ School of Nursing, Binzhou Medical University, Yantai, Shandong, China; ^2^ Department of Anesthesiology, Yantai Yu Huang Ding Hospital, Yantai, Shandong, China; ^3^ Medicine and Pharmacy Research Center, Binzhou Medical University, Yantai, Shandong, China; ^4^ Institute of Molecular Imaging, Binzhou Medical University, Yantai, Shandong, China

**Keywords:** prostate cancer, interleukin-10, polymorphism, peripheral blood interleukin-10, meta-analysis

## Abstract

This study purported to investigate the impact of interleukin-10 (IL-10) gene 4 polymorphisms (−1082G>A, -819T>C, -592A>C and 210T>C) on peripheral blood IL-10 variation and prostate cancer (PCa) risk, with a special consideration given to various origins of between-study heterogeneity. 2 researchers independently fulfilled literature retrieval, quality assessment and information collection. Sub-grouped analyses per ethnicity, continent, design type, control source, genotyping procedure, genotype validation, age-matched status, study sample size, quality score and controls’ mean age were conducted, respectively. Total 17 unduplicated studies (patients/controls: 7561/8101) were assessable for PCa risk, and 4 unduplicated studies (1189 subjects) for peripheral blood IL-10 variation. Pooling all assessable studies identified a marginally significant association between the -1082A allele and increased PCa risk (odds ratio (OR)=1.10, 95% confidence interval [CI]: 1.00 to 1.21) (Heterogeneity *I*^2^=64.3%), and no significance was detected in sub-grouped analyses of this polymorphism. Contrastingly, the -592C allele was significantly associated with reduced PCa risk in both prospective (OR=0.85, 95% CI: 0.77 to 0.95) and population-based (OR=0.92, 95% CI: 0.84 to 1.00) studies (Heterogeneity *I*^2^=0.0% and 18.1%). Moreover, carriers of combined -592CA/CC genotypes had a significant higher level of peripheral blood IL-10 than the -592AA genotype carriers (weighted mean difference=0.45 and 0.54 mg/dL, 95% CI: 0.23 to 0.67 and 0.30 to 0.39). The above comparisons possessed a low probability of publication bias. In sum, our findings suggested that IL-10 gene -592A>C polymorphism may represent a promising candidate locus for the occurrence of PCa, and further signified a contributing role of this polymorphism in prostate carcinogenesis.

## INTRODUCTION

Prostate cancer (PCa) is frequently occurring among men, and its mortality is continuing to rise, especially in some Western countries [1, 2]. As reported by the World Cancer Research Fund International (http://www.wcrf.org/int/cancer-facts-figures/data-specific-cancers/prostate-cancer-statistics), PCa represents 8 per cent of all new cancer cases and 15 per cent in men in 2012. The latest statistics from the American Cancer Society have predicted that PCa accounts for 21 per cent of all new cancer cases in men, and for 8 per cent of all male cancer deaths in the United States [3]. A growing body of evidence has suggested that chronic inflammation plays an important role in modulating cellular events during the course of prostate carcinogenesis through disrupting immune responses and altering tumor microenvironments [4, 5]. As an apt illustration of this evidence, the viability of PCa PC-3 cells was found to be significantly correlated with the ratio of pro-inflammatory to anti-inflammatory cytokines in macrophage-conditioned media [6]. Interleukin-10 (IL-10) is a key anti-inflammatory cytokine that can down-regulate pro-inflammatory responses [7]. IL-10 is secreted by Th2 cells, and its production is involved in the regulation of immune and inflammatory responses [8]. Based on the above evidence, it is tempting for us to assume a regulatory role of IL-10 in prostate carcinogenesis.

As we all know, PCa is a malignant cancer, and it exhibits familial aggregation [9]. Some studies have reported that there is a strong hereditary component in the development of PCa, which accounts for 5 to 10 per cent of all cases [10–12]. It is of crucial importance to pinpoint genetic determinants that can explain the interindividual differences in susceptibility to PCa. Given the promising candidacy of IL-10 in prostate carcinogenesis, a large number of investigators were inspired to hunt for IL-10 genetic alternations strongly associated with PCa susceptibility, while unfortunately their results remained indeterminate [13–17]. This may attribute to between-study distinctions in respect of ethnicity, design type, sample collection, statistical power and so forth. In this regard, two previous meta-analyses with the same purport published in 2011 [18, 19] have left some critical methodological questions unanswered, mainly revolving around the exploratory testing of between-study heterogeneity. We thereby decided to update the two meta-analyses with the incorporation of additional studies publicly available by November 2016. This paper purported to integrate the findings from 17 assessable studies to investigate the impact of IL-10 gene 4 polymorphisms on peripheral blood IL-10 variation and PCa risk. In the meantime, a special consideration was given to probe into various origins of between-study heterogeneity.

## RESULTS

### Study selection

**Figure 1:**
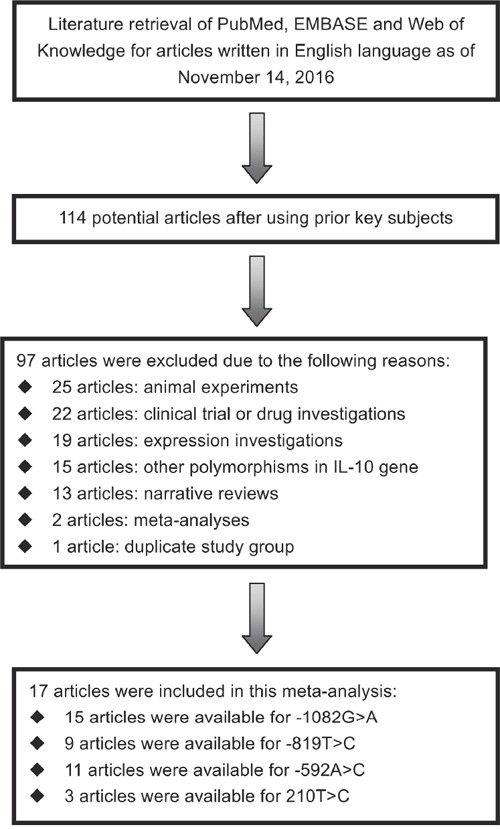
The streamline plot of article selection for the present meta-analysis

Literature retrieval of three public sources using prior key subjects identified a total of 114 articles written in the English language. Therein, 97 articles were debarred due to some precise reasons as elucidated in Figure 1. The rest 17 articles met our inclusive criteria, and were pooled consequently [13–17, 20–31]. The association of IL-10 gene 4 polymorphisms with PCa risk was examined in 16 articles [13–17, 20, 21, 23–31] incorporating 17 unduplicated studies (7561 patients and 8101 controls), and with peripheral blood IL-10 variation in 2 articles [21, 22] incorporating 4 unduplicated studies (1189 subjects). Table 1 shows the characteristics of 17 studies under investigation.

**Table 1:** The characteristics of 17 studies for the association of interleukin-10 genetic polymorphisms with prostate cancer risk

Author	Year	Region	Ethnicity	Design type	Control source	Genotyping procedure	Validation	Match	Quality score	Patients	Controls
Winchester	2015	USA	Caucasian	Prospective	Population	MassARRAY	YES	YES	11	881	848
Horvat	2015	Croatia	Caucasian	Prospective	Hospital	PCR-related	N.R.	YES	9	120	120
Dwivedi	2015	India	Asian	Retrospective	Hospital	PCR-related	YES	YES	8	291	291
Ianni	2013	Italy	Caucasian	Retrospective	Population	PCR-related	YES	N.R.	7	244	259
Dluzniewski	2012	USA	Mixed	Prospective	Population	MassARRAY	N.R.	YES	8	484	484
VanCleave	2010	USA	African-American	Retrospective	Hospital	TaqMan	YES	N.R.	10	193	666
Liu	2010	China	Asian	Retrospective	Population	PCR-related	YES	YES	9	262	270
Wang	2009	USA	Mixed	Prospective	Population	TaqMan	N.R.	YES	7	264	264
Kesarwani	2009	India	Asian	Retrospective	Hospital	PCR-related	YES	YES	9	159	259
Zabaleta (C)	2008	USA	Caucasian	Retrospective	Hospital	TaqMan	YES	N.R.	9	475	394
Zabaleta (AA)	2008	USA	African-American	Retrospective	Hospital	TaqMan	YES	N.R.	9	66	129
Omrani	2008	Iran	Asian	Retrospective	Hospital	PCR-related	N.R.	N.R.	6	41	103
Faupel-Badger	2008	Finland	Caucasian	Prospective	Population	TaqMan	YES	YES	9	584	584
Eder	2007	Austria	Caucasian	Retrospective	Population	TaqMan	YES	YES	7	547	545
Michaud	2006	USA	Mixed	Prospective	Population	TaqMan	YES	YES	11	1320	1842
Xu	2005	Sweden	Caucasian	Retrospective	Population	MassARRAY	N.R.	YES	8	1383	780
McCarron	2002	UK	Caucasian	Retrospective	Population	PCR-related	N.R.	N.R.	7	247	263

Abbreviations: PCR: polymerase chain reaction; N.R.: not reported.

8 of 17 studies enrolled Caucasians, 4 studies enrolled Asians, 2 studies enrolled African-Americans and 3 studies enrolled mixed-ethnicity populations of Caucasian and African-American. 11 studies followed retrospective design and 6 studies followed prospective design. 10 studies were population-based studies, and 7 studies were hospital-based studies. IL-10 genotypes were determined by TaqMan in 7 studies, by PCR-related methods in 7 studies and by MassARRAY in 3 studies. Only 11 studies validated the accuracy of genotypes, either internally or externally. Age was reported to be matched in frequency between PCa patients and controls in 11 studies. 9 studies possessed a total sample size of less than 600 (the median cut-off point of total sample sizes of all assessable studies), and 8 studies involved more than 600 subjects. Quality score of 17 studies ranged from 6 to 11, and its mean value was 8.47. To be specific, 15, 9, 11 and 3 studies were assessable to respectively investigate the impact of IL-10 gene -1082G>A, -819T>C, -592A>C and 210T>C polymorphisms on PCa risk.

### IL-10 genetic polymorphisms and PCa risk: Overall analyses

**Figure 2:**
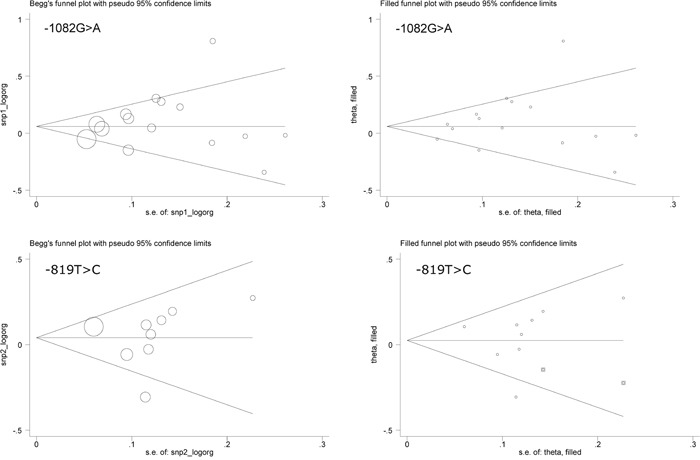
The Begg's (the left panels) and filled (the right panels) funnel plots for interleukin-10 gene -1082G>A and -819T>C polymorphisms under the allelic model

**Figure 3:**
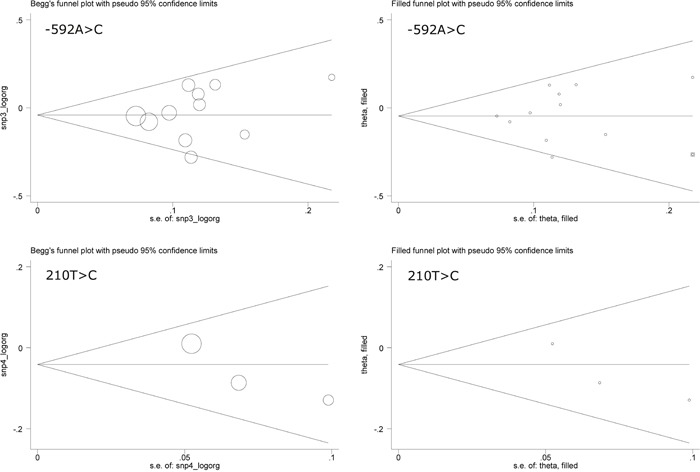
The Begg's (the left panels) and filled (the right panels) funnel plots for interleukin-10 gene -592A>C and 210T>C polymorphisms under the allelic model

The prediction of IL-10 gene each polymorphism for PCa risk was explored by comparing mutant allele with wild allele (termed the allele model), heterozygous genotype with wild genotype (termed the hetero-genotype model), homozygote genotype with wild genotype (termed the homo-genotype model) and combined heterozygote/homozygous genotypes with wild genotype (termed the dominant model), respectively. Overall risk prediction is offered in Table 2. The -1082A allele and the -1082AA genotype were marginally associated with increased PCa risk, with an OR of respectively being 1.10 (95% CI: 1.00 to 1.21) and 1.23 (95% CI: 1.01 to 1.50) (Heterogeneity *I*^2^=64.3% and 63.2%). No hints of significant association and between-study heterogeneity were observed for the other three polymorphisms under investigation. Both Begg's and Egger's tests indicated no statistical evidence of publication bias. The Begg's funnel plots seemed symmetrical for all 4 polymorphisms (Figures 2 and Figure 3). As reflected by the filled funnel plots (Figures 2 and Figure 3), there were respectively 2 studies and 1 study required for -819T>C and -592A>C polymorphisms respectively to render funnel plots symmetrical.

**Table 2:** Overall association of interleukin-10 gene 4 polymorphisms with prostate cancer risk under 4 genetic models

Genetic models	Num.	OR	95% CI	P	*I*^2^	P_Begg_	P_Egger_	Num. of missing studies	Filled OR, 95% CI, P
***Allele model***									
−1082G>A	15	1.10	1.00 to 1.21	0.056	64.3%	0.692	0.284	0	
−819T>C	9	1.04	0.94 to 1.15	0.490	46.8%	0.251	0.961	2	1.01, 0.92 to 1.11, 0.244
−592A>C	11	0.96	0.89 to 1.04	0.316	24.3%	0.553	0.470	1	0.96, 0.89 to 1.03, 0.235
210T>C	3	0.63	0.88 to 1.04	0.283	8.2%	0.296	0.254	0	
***Hetero-genotype model***									
−1082G>A	15	1.11	0.95 to 1.30	0.200	56.5%	0.621	0.483	0	
−819T>C	9	0.97	0.84 to 1.13	0.717	0.0%	0.466	0.523	0	
−592A>C	11	0.92	0.80 to 1.07	0.296	0.0%	0.350	0.351	0	
210T>C	3	0.97	0.86 to 1.10	0.657	0.0%	0.296	0.258	0	
***Homo-genotype model***									
−1082G>A	15	1.23	1.01 to 1.50	0.039	63.2%	0.488	0.232	0	
−819T>C	9	1.08	0.89 to 1.31	0.431	23.8%	0.602	0.740	1	1.06, 0.88 to 1.28, 0.626
−592A>C	11	0.92	0.78 to 1.07	0.261	0.0%	1.000	0.959	0	
210T>C	3	0.92	0.78 to 1.08	0.295	10.4%	0.296	0.255	0	
***Dominant model***									
−1082G>A	15	1.15	0.98 to 1.36	0.095	63.7%	0.921	0.434	0	
−819T>C	9	1.02	0.88 to 1.18	0.813	7.2%	0.175	0.498	0	
−592A>C	11	0.93	0.81 to 1.07	0.295	0.0%	0.213	0.312	0	
210T>C	3	0.96	0.85 to 1.08	0.456	0.0%	0.296	0.302	0	

Abbreviations: OR: odds ratio; 95% CI: 95 per cent confidence interval; *I*^2^: inconsistency index.

### IL-10 genetic polymorphisms and PCa risk: Sub-grouped analyses

As only 3 studies were assessable for 210T>C polymorphism, its association with PCa risk was unlikely to be of use in sub-grouped analyses. For the rest 3 polymorphisms, sub-grouped analyses were undertaken to probe into various origins of heterogeneity, per ethnicity, continent, design type, control source, genotyping procedure, genotype validation, matching status, study sample size, quality score (at the median cut-off point of 9) and controls’ mean age (> 50 years old), respectively (Table 3). For -1082G>A, there was marginal significance in Caucasians, when comparing the -1082A allele with the -1082G allele (OR=1.13, 95% CI: 0.97 to 1.32). Further by continental regions, the association of -1082A allele with PCa risk was strengthened in studies from European countries (OR=1.24, 95% CI: 1.01 to 1.54). In population-based studies, the -1082A allele was associated with a 1.15-fold increased PCa risk (95% CI: 1.02 to 1.31). Genotype misclassification might be a possible origin of heterogeneity, as significance was noticed after confining analysis to studies lacking genotype validation. In addition, in the studies with quality score ≤ 9, there was a marginally increased PCa risk for the comparison of the -1082A allele with the -1082G allele (OR=1.14, 95% CI: 1.00 to 1.29).

**Table 3:** Sub-grouped association of interleukin-10 gene 3 polymorphisms with prostate cancer risk

Subgroups		−1082G>A polymorphism					−819T>C polymorphism					−592A>C polymorphism				
		Num.	OR	95% CI	P	*I*^2^	Num.	OR	95% CI	P	*I*^2^	Num.	OR	95% CI	P	*I*^2^
***Ethnicity***	Caucasian	7	1.13	0.97 to 1.32	0.115	75.8%	3	0.92	0.73 to 1.15	0.474	70.8%	5	0.94	0.85 to 1.05	0.276	41.7%
	Asian	3	0.99	0.70 to 1.41	0.971	52.2%	3	1.09	0.94 to 1.26	0.238	0.0%	2	1.11	0.93 to 1.32	0.249	0.0%
	African-American	2	1.03	0.84 to 1.27	0.786	0.0%	2	1.11	0.90 to 1.37	0.317	0.0%	2	1.06	0.86 to 1.30	0.606	0.0%
	Mixed	3	1.13	0.91 to 1.40	0.282	78.8%	1	1.11	0.99 to 1.25	0.082	NA	2	0.84	0.71 to 1.00	0.051	0.0%
***Continent***	North America	7	1.04	0.94 to 1.15	0.486	53.0%	5	1.08	0.99 to 1.17	0.084	0.0%	6	0.96	0.87 to 1.07	0.468	16.7%
	Europe	5	1.24	1.01 to 1.54	0.044	75.9%	1	0.74	0.59 to 0.92	0.007	NA	3	0.90	0.78 to 1.04	0.155	44.3%
	Asia	3	0.99	0.70 to 1.41	0.971	52.2%	3	1.09	0.94 to 1.26	0.238	0.0%	2	1.11	0.93 to 1.32	0.249	0.0%
***Design type***	Prospective	6	1.08	0.97 to 1.20	0.167	53.4%	3	0.93	0.74 to 1.17	0.543	81.1%	4	0.85	0.77 to 0.95	0.003	0.0.%
	Retrospective	9	1.11	0.94 to 1.32	0.213	71.4%	6	1.10	0.99 to 1.23	0.067	0.0%	7	1.03	0.95 to 1.11	0.499	0.0%
***Control source***	Hospital	6	0.98	0.87 to 1.10	0.718	0.0%	5	1.09	0.97 to 1.23	0.129	0.0%	4	1.09	0.96 to 1.24	0.182	0.0%
	Population	9	1.15	1.02 to 1.31	0.026	75.0%	4	0.98	0.81 to 1.18	0.798	74.3%	7	0.92	0.84 to 1.00	0.041	18.1%
***Genotyping***	MassARRAY	3	1.08	1.00 to 1.18	0.052	0.0%	1	0.94	0.78 to 1.14	0.539	NA	3	0.92	0.83 to 1.01	0.078	0.0%
	PCR-related	6	1.18	0.89 to 1.58	0.254	74.3%	3	1.09	0.94 to 1.26	0.238	0.0%	2	1.11	0.93 to 1.32	0.249	0.0%
	TaqMan	6	1.03	0.91 to 1.17	0.626	55.5%	5	1.03	0.87 to 1.22	0.738	66.2%	6	0.96	0.84 to 1.10	0.571	41.5%
***Validation***	N.R.	6	1.15	1.04 to 1.27	0.004	12.7%	0					3	0.91	0.81 to 1.02	0.082	0.0%
	YES	9	1.07	0.93 to 1.23	0.353	72.2%	9	1.04	0.96 to 1.30	0.490	46.8%	8	1.00	0.90 to 1.10	0.914	33.0%
***Age-matched***	N.R.	6	1.16	0.89 to 1.52	0.263	79.0%	3	1.12	0.96 to 1.30	0.157	0.0%	3	1.09	0.94 to 1.27	0.243	0.0%
	YES	9	1.07	0.99 to 1.17	0.106	46.9%	6	1.01	0.88 to 1.15	0.940	62.5%	8	0.93	0.86 to 1.01	0.092	22.5%
***Sample size***	< 600	8	1.19	0.96 to 1.47	0.111	66.9%	4	1.11	0.97 to 1.28	0.135	0.0%	4	1.06	0.92 to 1.22	0.461	0.0%
	≥ 600	7	1.03	0.96 to 1.11	0.430	37.8%	5	0.99	0.86 to 1.15	0.902	65.5%	7	0.94	0.86 to 1.02	0.134	30.0%
***Quality score***	≤ 9	12	1.14	1.00 to 1.29	0.049	65.8%	6	1.04	0.88 to 1.24	0.641	60.3%	9	0.97	0.88 to 1.06	0.466	37.2%
	> 9	3	0.99	0.92 to 1.07	0.758	0.0%	3	1.06	0.96 to 1.14	0.246	4.8%	2	0.95	0.83 to 1.09	0.476	0.0%
***Controls’ age***	> 50 years	8	1.15	0.96 to 1.37	0.130	77.3%	4	1.12	1.02 to 1.23	0.017	0.0%	5	0.96	0.86 to 1.06	0.412	5.2%

Abbreviations: OR: odds ratio; 95% CI: 95 per cent confidence interval; *I*^2^: inconsistency index; PCR: polymerase chain reaction; N.R.: not reported.

For -819T>C, there was no observed significance in all subgroups, with an exception of the studies with controls’ mean age > 50 years old (OR=1.12, 95% CI: 1.02 to 1.23). For -592A>C, association of the -592C allele with reduced PCa risk was significant in the studies with mixed-ethnicity populations (Caucasians and African-Americans) (OR=0.84, 95% CI: 0.71 to 1.00), as well as in prospective studies (OR=0.85, 95% CI: 0.77 to 0.95) and population-based studies (OR=0.92, 95% CI: 0.84 to 1.00).

### IL-10 genetic polymorphisms and peripheral blood IL-10

**Figure 4:**
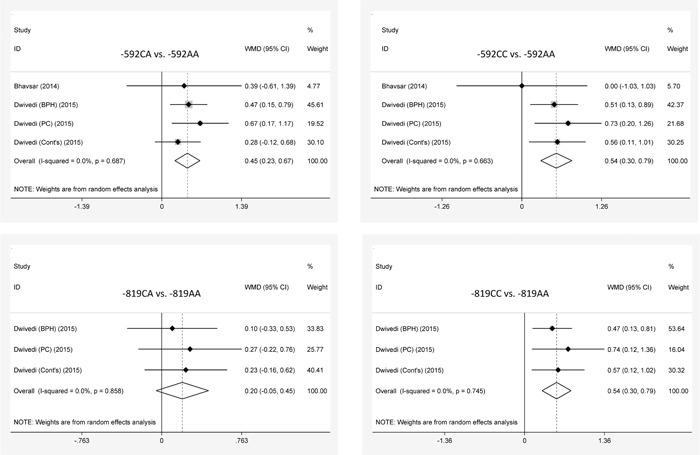
The forest plots for interleukin-10 gene -819T>C and -592A>C polymorphisms on peripheral blood interleukin-10 variation (mg/dL)

The characteristics of 4 studies for the association of interleukin-10 gene -592A/C and -819T>C polymorphisms with peripheral blood interleukin-10 variation are presented in Supplementary Table 2. Mean differences in peripheral blood IL-10 across the genotypes of -819T>C and -592A>C polymorphisms are displayed in Figure 4. Carriers of the -819CC genotype had a significant higher level of peripheral blood IL-10 than the -819TT genotype carriers (WMD=0.54 mg/dL, 95% CI: 0.30 to 0.79), which no significance was noticed between carriers of the -819CT genotype and the -819TT genotype. For -592A>C, carriers of the -592CA and the -592CC genotypes had a significant higher level of peripheral blood IL-10 than the -592AA genotype carriers (WMD=0.45 and 0.54 mg/dL, 95% CI: 0.23 to 0.67 and 0.30 to 0.39). There was no heterogeneity for above comparisons (*I*^2^=0.0%).

### Meta-regression analysis

After taking age, per cent of smokers, family PCa history and PSA as independent variables, a meta-regression model was constructed. Unfortunately, none of these 4 variables showed a significant impact on the association of IL-10 gene 4 polymorphisms with PCa risk (P>0.05).

## DISCUSSION

In the present study, we meta-analytically investigated the impact of IL-10 gene 4 polymorphisms on peripheral blood IL-10 variation and PCa risk. The most important observation was that the mutation of -592A>C polymorphism was significantly associated with reduced PCa risk and increased peripheral blood IL-10 level. Moreover, ethnicity, design type and control source served as the possible origins of heterogeneity. As the -592A>C polymorphism is in IL-10 gene promoter region, it appears reasonable to postulate that this polymorphism may alter an individual's susceptibility to PCa risk by regulating peripheral blood IL-10 level.

As indicated by two previous meta-analyses, there were no signs of statistical significance between IL-10 gene 3 widely-studied promoter polymorphisms (−1082G>A, -819T>C and -592A>C polymorphisms) and PCa risk under all possible genetic models of inheritance [18, 19]. By contrast, in this updated meta-analysis, we integrated the results of 17 articles and found that IL-10 gene -592A>C polymorphism was significantly associated with PCa risk in both prospective and population-based studies. In addition, we observed a significant relation between -592A>C polymorphism and peripheral blood IL-10 variation, which can lend some credence to the functional role of this polymorphism in prostate carcinogenesis. In support of this claim, the -592C allele was reported to be associated with a higher level of IL-10 mRNA expression [32], in some kind of agreement with the findings from the present meta-analysis. Moreover, a five-year follow-up study indicated that the CC genotype of -592A>C polymorphism in association with higher relative mRNA expression exhibited a decreased chance of survival among PCa patients [33]. We therefore developed a working hypothesis that the -592A>C polymorphism may serve as a candidate genetic marker predictive of risk for PCa by altering IL-10 expression.

Differing from previous meta-analyses, we in this meta-analysis comprehensively probed into potential origins of between-study heterogeneity by both sub-grouped analyses and meta-regression analyses. Our findings suggested that ethnicity might be a potential origin of heterogeneity, in view of discrepant estimates between IL-10 genetic polymorphisms and PCa risk. It is easily understood that linkage pattern in human genomes varies during evolution in populations of different ethnic backgrounds [34], which, at least in part, can interpret the wide differences in the frequency of PCa across ethnicities. Currently, PCa is one of the most common malignancies in Western countries and an emerging malignancy in developing nations [35]. Besides the differences in styles and standards of living, studies in families have suggested that PCa is genetically determined [36]. The genetic basis of PCa is complex and currently not fully understood [37, 38]. IL-10 as a potential functional candidate needs further *in-vitro* and *in-vivo* experimental investigations. On the other hand, we also found that design type and control source were the other two potential contributors interpreting between-study heterogeneity in the present meta-analysis. It is widely accepted that case-control studies are often prone to selection bias that often occurs, if the cases are not representative of all cases within the population and the controls are not representative of the population where the cases are from [39, 40]. It is worth noting that our further sub-grouped analyses found that association of the -592A>C polymorphism with PCa risk was strongly potentiated in both prospective and population-based studies, indicative of the robustness of our findings. Nevertheless, considering insufficient statistical power in sub-grouped analyses, we should underscore the necessity for future replication studies with a larger sample size.

Several limitations of the present meta-analysis deserved comments. Firstly, the probability of publication bias was always a concern for a meta-analysis [41], because our results were merely geared on published articles from the English-language journals. In addition, our filled funnel plots suggested missing studies for -819T>C and -592A>C polymorphisms, while no statistical significance was detected by both Begg's and Egger's tests. Nonetheless, we were unable to exclude the possible existence of publication bias merely based on statistical tests, which can be minimized by incorporating more and more such studies. Secondly, the limited selection of IL-10 genetic polymorphisms was another concern, because only 4 polymorphisms were investigated in association with PCa risk. Thirdly, the interaction of IL-10 genetic polymorphisms with environmental factors was unable to be explored, because a prospective study found that IL-10 gene promoter polymorphisms interacting with tobacco exposure may alter the susceptibility risk and severity of PCa [42]. Fourthly, other origins of between-study heterogeneity remained an open question, such as diets, lifestyles, physical activity and so forth, because these data were unavailable for us. Fifthly, most studies in this meta-analysis enrolled subjects from Western countries, and it was premature to extrapolate our findings to the other races or ethnicities.

In sum, our meta-analytical findings suggested that IL-10 gene -592A>C polymorphism may represent a promising candidate locus for the occurrence of PCa, and further signified a contributing role of this polymorphism in prostate carcinogenesis. Further studies covering more genome regions on IL-10 gene and related inflammatory genes are warranted among diverse ethnic groups to gain a deeper insight into the impact of IL-10 genetic alterations on peripheral blood IL-10 variation and PCa risk, which can aid in dissecting the molecular mechanisms of PCa for further therapeutic targeting.

## MATERIALS AND METHODS

### Study protocol

This meta-analysis is geared on genetic observational studies. The conduct of this meta-analysis methodologically accords with the chief protocols of the Meta-analysis Of Observational Studies in Epidemiology (MOOSE) statement [43].

### Literature retrieval

Retrieval of published articles was confined to electronic sources of Medline (PubMed), EMBASE (Excerpt Medica Database) and Web of Knowledge. Key subjects for retrieval consisted of (“prostate cancer” or “prostate carcinoma”) in the Title and (“interleukin-10” or “interleukin 10” or “IL-10” or “cytokine” or “inflammatory” or “inflammation”) in the Title/Abstract and (“genetic” or “polymorphism” or “variant” or “mutation” or “allele” or “genotype” or “circulating” or “plasma” or “serum” or “peripheral blood”) in the Abstract. The last retrieval date was November 14, 2016. Articles written in non-English languages were ignored. Citations of retrieved articles and previous meta-analyses were also inspected. Literature retrieval was fulfilled by 2 researchers (Dan Wang and Fang Liu).

### Inclusive criteria

As the purport of this meta-analysis was to investigate the impact of IL-10 gene all potential polymorphisms on peripheral blood IL-10 variation and PCa risk, articles were includable if they met the three criteria simultaneously, as follows: (i) study subjects were composed of human adults (aged 18 years old or over); (ii) at least one polymorphism in IL-10 gene was determined by standard methods, and only the polymorphism assessed by three or more independent studies was meta-analyzed; (iii) the genotypes or if unavailable the alleles of assessed polymorphism(s) were delivered between PCa patients and controls or mean peripheral blood IL-10 level along with its standard deviation were delivered across genotypes of the polymorphism under investigation in PCa patients or controls or both. According to the 2^nd^ inclusive criteria, 4 polymorphisms absorbed our attention in the present study, and they were -1082G>A (rs1800896), -819T>C (rs1800871), -592A>C (rs1800872) and 210T>C (rs3024496).

### Quality appraisal

Quality of the articles that met above-listed inclusive criteria was justified by producing a quality score based on 7 respects of a genetic association study (more details seen in Supplementary Table 1). This quality score was coined by Thakkinstian et al. in 2005 [44], and its range spanned from 0 (the worst) to 12 (the best). Quality appraisal was implemented independently by 2 researchers who were responsible for literature retrieval, and a discussion was made until every respect was entirely consistent by comparison.

### Information collection

Anthropometric, clinical and genetic data were excerpted from each assessable study in this meta-analysis by 2 researchers (Dan Wang and Fang Liu), as follows: family name of the first author, publishing year, sampling region, ethnicity, design type, control source (hospital-based and population-based), genotyping procedure, (internal - using the same genotyping method or external - using a different genotyping method in some randomly-selected samples) genotype validation, age-matched status, study sample size, mean age, per cent of current or ever smokers, family PCa history, prostate specific antigen (PSA), the genotypes of 4 above-listed polymorphisms between PCa patients and controls, mean (standard deviation) of peripheral blood IL-10 level in PCa patients or controls or both. Consistency of data excerpted by the 2 researchers was tested with any disagreement resolved through discussing.

### Statistical analysis

The impact of IL-10 gene 4 polymorphisms on peripheral blood IL-10 variation and PCa risk was respectively signified with weighted mean difference (WMD) and odds ratio (OR), along with 95 per cent confidence interval (95% CI) under either a fixed-effects model in case of non-significant heterogeneity or a random-effects model in otherwise case. Significance of between-study heterogeneity was connoted by the *I*^2^, the acronym for inconsistency index, which characterized the per cent of variability due to heterogeneity rather than due to chance. Heterogeneity is judged significant if the *I*^2^ overpasses 50 per cent [45]. To probe into potential origins of heterogeneity, sub-grouped analyses per ethnicity, continent, design type, control source, genotyping procedure, genotype validation, age-matched status, study sample size, quality score and controls’ mean age (> 50 years old) were respectively applied. In addition, meta-regression analyses were applied with embracing age, per cent of smokers, family PCa history and PSA as independent variables to further interpret additional origins of between-study heterogeneity. Publication bias was graphically judged from the Begg's and filled funnel plots, along with the Begg's and Egger's regression tests. All statistical analyses were achieved by aid of the Stata software version 11.0 for the Windows 10.0.

## SUPPLEMENTARY TABLES



## Abbreviations

IL-10: interleukin-10
PCa: prostate cancer
PCR: polymerase chain reaction
OR: odds ratio
95% CI: 95 per cent confidence interval
WMD: weighted mean difference
*I*^2^: inconsistency index
MOOSE: Meta-analysis Of Observational Studies in Epidemiology
EMBASE: Excerpt Medica Database
