# Supplementary material for: The impact of interleukin-10 (IL-10) gene 4 polymorphisms on peripheral blood IL-10 variation and prostate cancer risk based on published studies
Source: Oncotarget. 2017 Apr 29;8(28):45994–6005. doi: 10.18632/oncotarget.17522 (PMC5542243; doi:10.18632/oncotarget.17522)
Supplement: Supplementary file 1 [file oncotarget-08-45994-s001.pdf]

The impact of interleukin-10 (IL-10) gene 4 polymorphisms on peripheral blood IL-10 variation and prostate cancer risk based on published studies

SUPPLEMENTARY MATERIALS

Supplementary Table 1: Quality appraisal criteria of interleukin-10 genetic polymorphisms with prostate cancer risk

Criteria	Quality score
Representativeness of cases	
A. Consecutive/randomly selected from case population with clearly defined random frame	2
B. Consecutive/randomly selected from case population without clearly defined random frame or with extensive inclusion criteria	1
C. Method of selection not described	0
Representativeness of controls	
D. Controls were consecutive/randomly drawn from the same area (ward/community) as cases with the same criteria	2
E. Controls were consecutive/randomly drawn from a different area than cases	1
F. Not described	0
Ascertainment of prostate cancer cases	
G. Clearly described objective criteria for diagnosis of prostate cancer	1
H. Not described	0
Ascertainment of controls	
I. Clinical examinations were performed on controls to prove that controls did not have prostate cancer	2
J. Article merely stated that controls were subjects who did not have prostate cancer; no proof provided	1
K. Not described	0
Ascertainment of genotyping examination	
L. Genotyping done under "blind" conditions	1
M. Unblended or not mentioned	0
Test for Hardy-Weinberg equilibrium	
N. Hardy-Weinberg equilibrium in control group	2
O. Hardy-Weinberg disequilibrium in control group	1
P. Hardy-Weinberg equilibrium not checked	0
Association assessment	
Q. Assessed association between interleukin-10 genotypes and prostate cancer with appropriate statistic and adjusting confounders	2
R. Assessed association between interleukin-10 genotypes and prostate cancer with appropriate statistic without adjusting confounders	1
S. Inappropriate statistic used	0

Supplementary Table 2: The characteristics of 4 studies for the association of interleukin-10 gene -592A/C and -819T>C polymorphisms with peripheral blood interleukin-10 variation

Peripheral blood interleukin-10 level (mg/dL) across -592A>C genotypes								
**First author**	**Year**	**Study subjects**	**N (-592CC)**	**Mean (-592CC)**	**N (-592CA)**	**Mean (-592CA)**	**N (-592AA)**	**Mean (-592AA)**
Bhavsar	2014	Controls	315	2.43	179	2.82	19	2.43
Dwivedi (BPH)	2015	BPH	30	3.53	38	3.49	26	3.02
Dwivedi (PCa)	2015	PCa patients	110	12.53	125	12.47	56	11.8
Dwivedi (Cont's)	2015	Controls	98	2.89	138	2.61	55	2.33
Peripheral blood interleukin-10 level (mg/dL) across -819T>C genotypes								
**First author**	**Year**	**Study subjects**	**N (-819CC)**	**Mean (-819CC)**	**N (-819CT)**	**Mean (-819CT)**	**N (-819TT)**	**Mean (-819TT)**
Bhavsar	2014	Controls	NA	NA	NA	NA	NA	NA
Dwivedi (BPH)	2015	BPH	29	3.53	24	3.16	41	3.06
Dwivedi (PCa)	2015	PCa patients	68	12.54	131	12.07	92	11.8
Dwivedi (Cont's)	2015	Controls	60	2.86	151	2.52	80	2.29

Abbreviations: PCa: prostate cancer; BPH: benign prostate hypertrophy; Cont's: controls; NA: not available.
